# Propofol Protects the Immature Rabbit Heart against Ischemia and Reperfusion Injury: Impact on Functional Recovery and Histopathological Changes

**DOI:** 10.1155/2014/601250

**Published:** 2014-08-27

**Authors:** Makoto Shirakawa, Hajime Imura, Takashi Nitta

**Affiliations:** ^1^Department of Cardiovascular Surgery, Nippon Medical School, 1-1-5 Sendagi, Bunkyo-ku, Tokyo 113-8603, Japan; ^2^Department of Cardiovascular Surgery, Nippon Medical School Musashikosugi Hospital, 1-396 Kosugicho, Nakahara-ku, Kawasaki, Kanagawa 211-8533, Japan

## Abstract

The general anesthetic propofol protects the adult heart against ischemia and reperfusion injury; however, its efficacy has not been investigated in the immature heart. This work, for the first time, investigates the cardioprotective efficacy of propofol at clinically relevant concentrations in the immature heart. Langendorff perfused rabbit hearts (7–12 days old) were exposed to 30 minutes' global normothermic ischemia followed by 40 minutes' reperfusion. Left ventricular developed pressure (LVDP) and coronary flow were monitored throughout. Lactate release into coronary effluent was measured during reperfusion. Microscopic examinations of the myocardium were monitored at the end of reperfusion. Hearts were perfused with different propofol concentrations (1, 2, 4, and 10 *μ*g/mL) or with cyclosporine A, prior to ischemic arrest and for 20 minutes during reperfusion. Propofol at 4 and 10 *μ*g/mL caused a significant depression in LVDP prior to ischemia. Propofol at 2 *μ*g/mL conferred significant and maximal protection with no protection at 10 *μ*g/mL. This protection was associated with improved recovery in coronary flow, reduced lactate release, and preservation of cardiomyocyte ultrastructure. The efficacy of propofol at 2 *μ*g/mL was similar to the effect of cyclosporine A. In conclusion, propofol at a clinically relevant concentration is cardioprotective in the immature heart.

## 1. Introduction

Generation of reactive oxygen species and Ca^2+^ overload are key triggers of ischemia and reperfusion (I/R) injury of the heart and it is now generally agreed that their effect is due to opening of the mitochondrial permeability transition pore (MPTP) [1, 2]. Further evidence in support of a role for MPTP comes from work showing that inhibition of MPTP opening (e.g., with cyclosporine A (CsA)) is cardioprotective in vitro [1, 3, 4] and in vivo [5].

Propofol is a general anesthetic that is widely used for induction and maintenance of anesthesia during cardiac surgery and in postoperative sedation [6–8]. It has been shown to protect hearts against cardiac insults in a variety of experimental models [9–12]. Propofol can act as a free radical scavenger [13], enhance tissue antioxidant capacity [14, 15], and also inhibit calcium channels [16, 17]. Its antioxidant property could be responsible for its inhibitory action on MPTP opening in the Langendorff perfused rat heart [11] and for its antiapoptotic properties [18]. Its cardioprotective properties have clinical relevance as shown in work involving a pig model of cardiopulmonary bypass and intermittent antegrade warm blood cardioplegic arrest [19]. However, these beneficial effects of propofol have not been shown in immature rabbit hearts.

Vulnerability of the immature heart to cardiac insults remains controversial. This is largely due to the fact that the extent of dysfunction and injury is directly related to disruption to cellular metabolic and ionic homeostasis, which both change during development [3]. In addition to issues relating to vulnerability, there are also controversies surrounding the efficacy of different cardioprotective strategies [20–22]. For example, inhibiting MPTP can be protective in piglets [23] but not in neonatal 7-day-old rat hearts [24]. Thus, myocardial protection for immature hearts needs further investigation to catch up that of the mature heart.

To our best of knowledge we, for the first time, investigate the efficacy of propofol on immature hearts at clinically relevant concentrations in the present study. The aim of the current study was to test the hypothesis that, in addition to its reported ability to protect normal and diseased adult hearts, propofol would also protect immature hearts against I/R. To address this issue, we investigated the cardioprotective efficacy of different concentrations of propofol and compared it to inhibition of MPTP using CsA in an immature rabbit heart model.

## 2. Material and Methods

This study was approved by the Animal Ethics Committee of Nippon Medical School. All animals received care in compliance with the Principles of Laboratory Animal Care.

### 2.1. Animals and Heart Perfusion

Newborn rabbits (7–12 days old) were obtained from a commercial breeder (Saitama Experiment Animal, Saitama, Japan) and anesthetized with sodium pentobarbital (50 mg/kg) intraperitoneally. Hearts were removed and placed in cold (4°C) Krebs-Henseleit buffer solution (standard perfusion media). The composition of the Krebs-Henseleit buffer solution (in mM) is as follows: NaCl: 118.5, NaHCO_3_: 25.0, KCl: 4.75, MgSO_4_·7H_2_O: 1.19, KH_2_PO_4_: 1.18, Glucose: 11.0, pH: 7.4 (after gassed with 95% O_2_ and 5% CO_2_), and CaCl_2_·2H_2_O: 1.8. The ascending aorta was then quickly cannulated (within 20 seconds) and the heart was perfused in the Langendorff mode at 39.0°C [25] and constant perfusion pressure of 43 mmHg (which is equivalent to the mean aortic pressure for the age of the rabbits at the time of study) [25].

### 2.2. Experimental Protocols

**Scheme 1:**
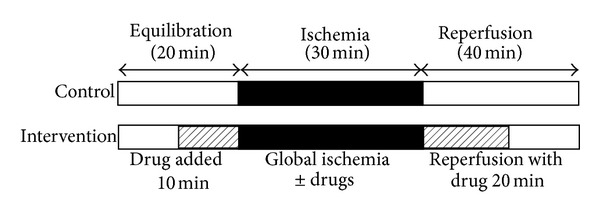
Experimental protocols for control and for intervention groups.

Details of the experimental protocols are shown in Scheme 1. After 10 minutes of stabilization, the heart was perfused for 10 minutes with normothermic standard perfusion media either with 2 *μ*g/mL intralipid which is the propofol vehicle (Fresemius Kabi Japan Inc, Tokyo, Japan) or with various concentrations of propofol (1, 2, 4, and 10 *μ*g/mL, *n* = 6 each). Diprivan (Astrazeneca Inc, Osaka, Japan) was the commercial formulation for propofol. After stabilization with or without the drug, the perfusion was stopped and normothermic global ischemia was commenced for 30 minutes. During this period, the hearts were immersed in buffer and maintained at 39.0°C in a temperature-controlled chamber. After the global ischemic period, the hearts were reperfused with the same preischemic media for 20 minutes after which the heart was perfused with buffer devoid of added propofol or intralipid.

In addition to investigating the protective effect of propofol, we also investigated the effect of the MPTP inhibitor, CsA. In these experiments, 0.2 *μ*mol/L of CsA was used.

### 2.3. Hemodynamic Measurement

The left ventricular pressure was monitored continuously with a handmade water-filled vinyl balloon, which was inserted into the left ventricle via the left atrial appendage and connected to pressure transducer as described previously [26]. The balloon volume was set so that an initial left ventricular end diastolic pressure (LVEDP) between 4 and 8 mmHg was achieved. The left ventricular developed pressure (LVDP) was calculated as the difference between left ventricular end systolic pressure and LVEDP.

### 2.4. Collection of Coronary Effluent

Coronary effluent was collected in an Eppendorf-tube before ischemia and at 0, 1, 3, and 5 minutes after reperfusion. The aliquots were frozen in liquid nitrogen and stored at −80°C. Lactate concentration in the coronary effluents was measured using Lactate Assay Kit (BioVision Research Products, CA, USA).

### 2.5. Collection and Histopathological Examination of Left Ventricular Myocardium

At the end of the protocols using propofol concentrations 0 (control), 2, and 10 *μ*g/mL, the free wall of the left ventricle was resected and preserved in 2% glutaraldehyde solution for histopathological investigation. All samples were investigated by an experienced pathologist (blinded to the protocols) using light and electron microscopy and evaluated by a semiquantitative method. In the assessment, each sample was simply marked as “a” (2 *μ*g/mL), “b” (control), or “c” (10 *μ*g/mL) without details of the protocols. The pathologist investigated all the samples (6 hearts from each group) in the same way as his previous report [27] and gave us his final results for each group. The scale of 0 to 4 represents the degrees of each morphological change and corresponds with the grading in his previous study [27]: 0, normal (grade 0); 1, trivial (grade 1); 2, mild (grade 2); 3, moderate (grade 3); and 4, severe (grades 4 and 5).

### 2.6. Statistical Analysis

Statistical analyses were performed using SPSS 16.0J for Windows (SPSS Japan Inc, Tokyo, Japan). Data were expressed as the mean ± SE unless otherwise stated. ANOVA was used to compare different groups and considered significant at *P* < 0.05 tested using Fisher's PLSD. Area under the curve to estimate total lactate release during reperfusion was calculated using the trapezium rule (Microsoft Excel).

## 3. Results

### 3.1. Effect of Different Concentrations of Propofol on Basal Hemodynamic Parameters

**Figure 1:**
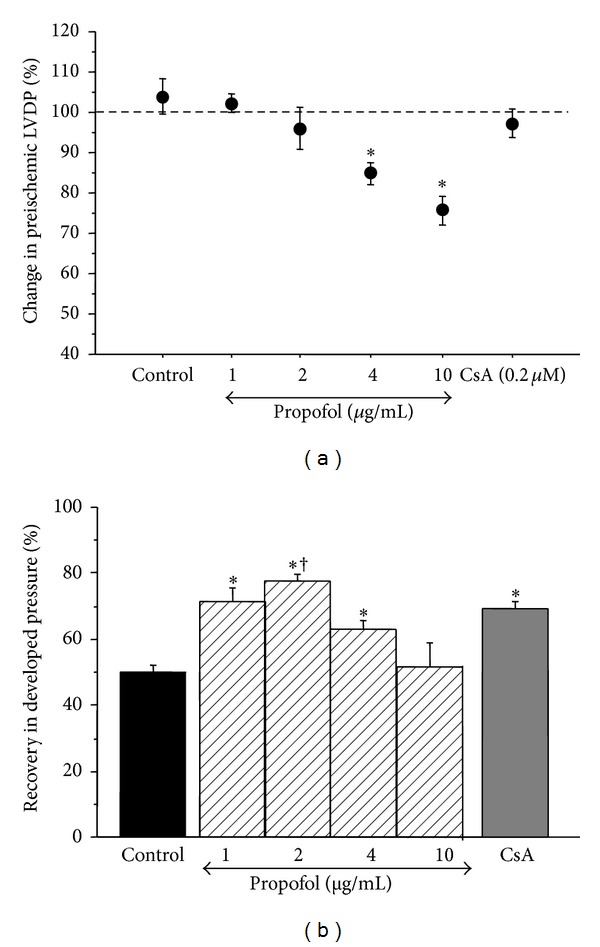
The effect of propofol on developed pressure. (a) Changes in left ventricular developed pressure prior to ischemia. The data shows the percentage change during 10 min perfusion with different concentrations of propofol or with cyclosporine A. Data are presented as mean ± SE. *Significant difference from basal level for the same intervention. (b) Recovery in developed pressure after ischemia and reperfusion. The data shows the change in LVDP at the end of protocol compared to basal line level. Data expressed as percentage of LVDP at the end of reperfusion/LVDP prior to ischemia. Data are presented as mean ± SE. *P < 0.05 versus control and 10 *μ*g/mL; ^†^ *P* < 0.05 versus 4 *μ*g/mL.

Figure 1(a) shows the effect of perfusion for 10 minutes with different concentrations of propofol or CsA on LVDP. Propofol at the higher concentrations of 4 or 10 *μ*g/mL caused a significant drop in LVDP. Propofol at lower concentrations or the addition of CsA did not alter LVDP.

### 3.2. Effect of Propofol and Cyclosporine A on Functional Recovery following I/R

Table 1(a) shows the values for LVDP and LVEDP measured before ischemic arrest, at the end of ischemia, and at the end of reperfusion. It is evident that the extent of the changes in both parameters was lower in groups perfused with low propofol or CsA. The percentage change (recovery) in LVDP for all groups is shown in Figure 1(b). Propofol at 2 *μ*g/mL showed the best recovery whilst 10 *μ*g/mL was not protective. Recovery with CsA was similar to 1 and 2 *μ*g/mL propofol.

### 3.3. Effect of Propofol and Cyclosporine A on Recovery in Coronary Flow following I/R

Changes in coronary flow for all groups before ischemia and at the end of the protocol are shown in Table 1(b). Propofol at 1 and 2 *μ*g/mL resulted in a significant improvement in coronary flow recovery compared to the control. On the other hand, both 4 and 10 *μ*g/mL propofol did not improve recovery. Recovery with CsA was between low and high propofol concentrations but did not reach statistical significance against control (*P* = 0.08).

### 3.4. Effect of Propofol on Lactate Release during Reperfusion

**Figure 2:**
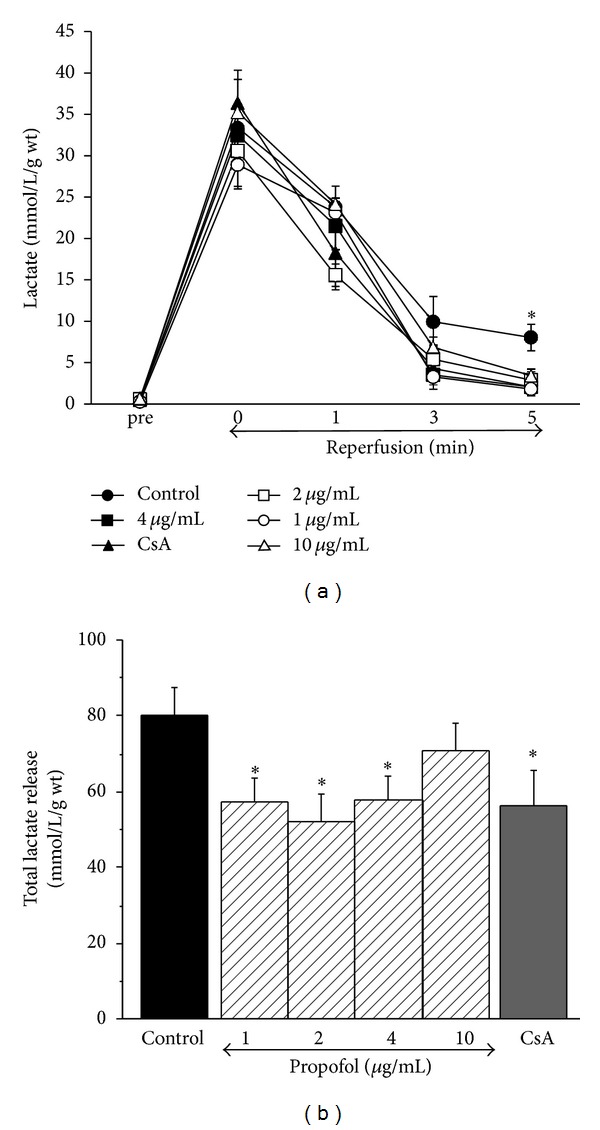
Lactate release during reperfusion. (a) Time-dependent lactate release. There were no significant differences between the groups at 0, 1, and 3 minutes after reperfusion (*P* = 0.09 at 3 minutes), whereas control group showed significantly higher lactate release than all other groups at 5 minutes of reperfusion. (b) Total lactate release during first 5 min of reperfusion. The data shows the total release of lactate as measured in the effluent during first 5 min of reperfusion using different interventions. Data are presented as mean ± SE. *P < 0.05 versus control.

Figure 2 shows time-dependent changes in lactate release and total lactate release as measured in coronary effluents during reperfusion for different interventions. All groups demonstrated similar changes with no significant differences for the first 3 minutes during reperfusion; however, control group showed significantly higher lactate release at 5 minutes compared to other groups (Figure 2(a)). Total lactate release was significantly lower than control for 1, 2, and 4 *μ*g/mL propofol (Figure 2(b)). It was also significantly lower for CsA. There was no significant difference between control and 10 *μ*g/mL propofol.

### 3.5. Histopathological Changes during I/R

The results for histopathological changes using light and electron microscopic studies are presented in Table 2. In summary, the following myocardial histological changes resulting from I/R were monitored: clumping of nuclear chromatin, intracellular and interstitial (extracellular) edema, appearance of abnormal I-band in myocytes (which are seen as white bands in myofibrils following ischemic damage), appearance of dense material deposit in mitochondria, clarity of mitochondrial matrix, and deformation of cristae [27].

#### 3.5.1. Light Microscopy Examination

**Figure 3:**
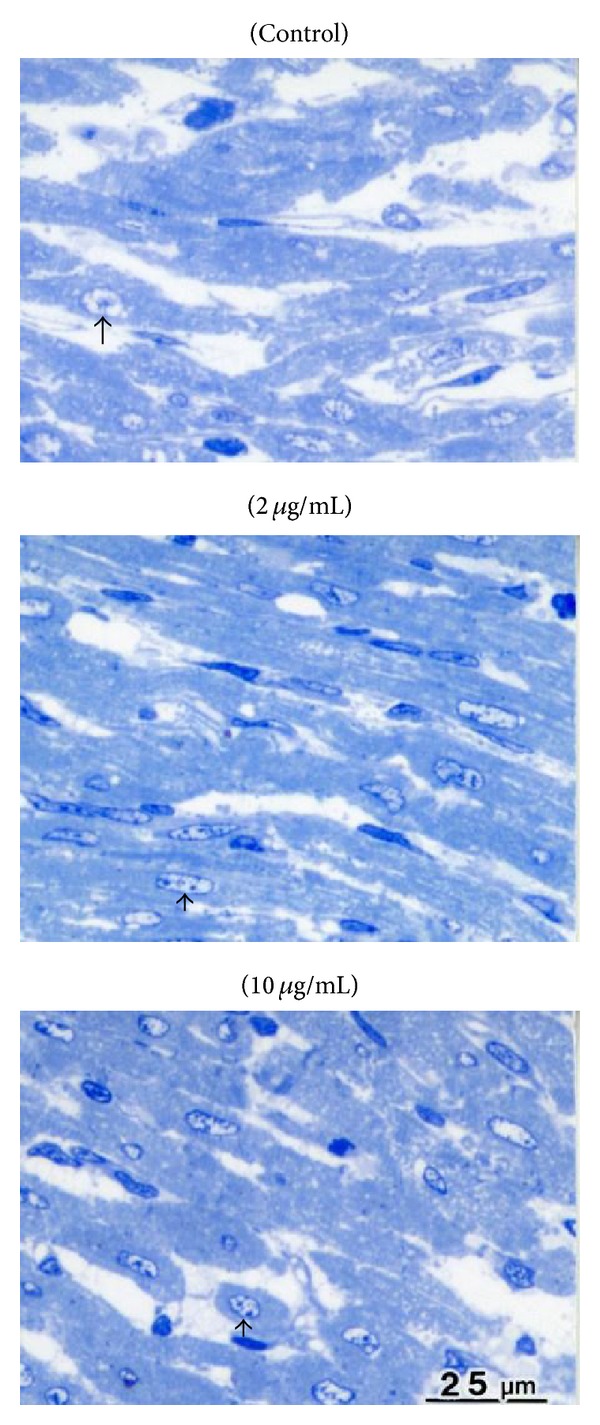
Histopathological assessment using light microscopy following ischemia and reperfusion. Light microscopy examination stained with toluidine blue (upper: control, middle: 2 *μ*g/mL of propofol, and lower: 10 *μ*g/mL of propofol). Myocardium with 2 *μ*g/mL of propofol showed mild intracellular swelling and clumping of nuclear chromatin, whilst these changes were more evident in myocardium with 0 and 10 *μ*g/mL of propofol. Interstitial (extracellular) edema was also less in 2 *μ*g/mL than in 0 and 10 *μ*g/mL.

The extent of the changes in nuclear morphology and intracellular edema was evaluated and tended to be lower in 2 *μ*g/mL of propofol compared to both control and 10 *μ*g/mL of propofol (Figure 3). While 2 *μ*g/mL of propofol was accompanied with mild or less interstitial (extracellular) edema, myocardium with 0 and 10 *μ*g/mL of propofol showed moderate or more severe interstitial (extracellular) edema after the protocols (Table 2).

#### 3.5.2. Electron Microscopy Examination

**Figure 4:**
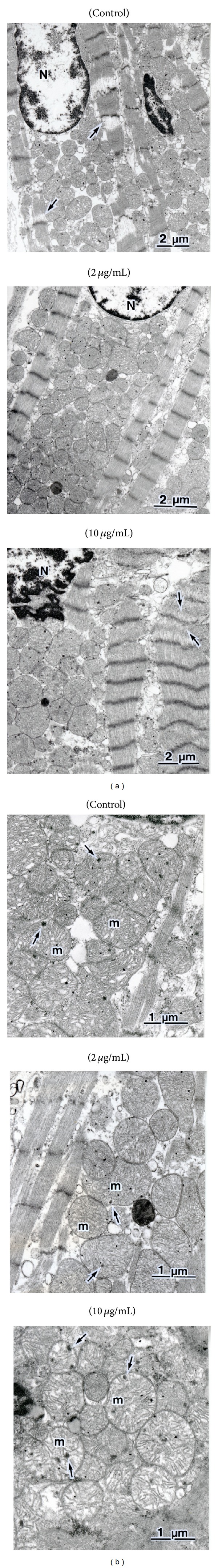
Electron micrographs following ischemia and reperfusion. (a) Electron micrographs of cardiomyocyte in each group (upper: control, middle: 2 *μ*g/mL of propofol, and lower: 10 *μ*g/mL of propofol). Many wide I-bands (arrows) appeared in the myocyte with 0 and 10 *μ*g/mL of propofol, whereas the I-band was not observed in 2 *μ*g/mL of propofol. (b) Dense material deposits (arrows) in mitochondria appeared in all groups (upper: control, middle: 2 *μ*g/mL of propofol, and lower: 10 *μ*g/mL of propofol). However, the size and number of these deposits were less in mitochondria with 2 *μ*g/mL of propofol than the other 2 groups. Moreover, structure of mitochondrial cristae was well preserved in group of 2 *μ*g/mL, whilst it was significantly deformed with separation in other 2 groups.

Clumping of nuclear chromatin, appearance of I-bands in myocytes, and mitochondrial changes (swelling and dense material deposit) tended to be lower in 2 *μ*g/mL propofol compared to control and to 10 *μ*g/mL propofol (Figures 4(a) and 4(b)).

## 4. Discussion

Propofol is widely used for anesthesia in current clinical practice and its target blood concentration is between 1 and 5 *μ*g/mL [28]. The cardioprotective potential of propofol against I/R injury in mature hearts is well established within this range in both clinical studies [29–31] and experimental models of normal [11, 19] and hypertrophic adult hearts [12]. It is known that the relevant blood concentration of propofol is similar for pediatric populations [32]; however, its cardioprotective effect has not been investigated. This study tested the hypothesis that propofol at a clinically relevant dose would enhance the functional recovery of the isolated immature heart following an ischemic insult. In addition, the protective potential of propofol in comparison to the MPTP inhibitor CsA was investigated. The results showed that after 30 minutes' global normothermic ischemia, propofol significantly improved functional recovery of LVDP and coronary flow (Figure 1(b) and Tables 1(a) and 1(b)). The cardioprotective efficacy of propofol was dose dependent and 2 *μ*g/mL of propofol showed the best functional recovery. Although 1 and 4 *μ*g/mL of propofol significantly improved functional recovery their beneficial effects were less than that of 2 *μ*g/mL. On the other hand, the functional recovery in the presence of 10 *μ*g/mL was no different from control (Figure 1(b)). Recovery in coronary flow was also improved with 2 and 1 *μ*g/mL propofol but not with higher concentrations. Finally, propofol at 2 *μ*g/mL reduced ultrastructural disruptions of cardiomyocytes caused by I/R (Figures 3 and 4 and Table 2).

Addition of propofol under baseline conditions caused a dose-dependent depression of LVDP where doses at 4 and 10 *μ*g/mL resulted in a significant fall compared to initial levels (Figure 1(a)). The depressive effects of propofol at high concentrations could be due to its known inhibitory effect of calcium channels [17, 33–35]. Taken together the current study supports a cardioprotective role for propofol in the immature heart exposed to I/R.

Several mechanisms have been proposed to explain the cardioprotective properties of propofol which include increased phosphorylation of phospho-glycogen-synthase kinase (GSK-3*β*) [36], inhibition of MPTP opening [11], reduced lipid peroxidation and lowered inflammation [37], and modulation of calcium channel activity [17]. The reduction in levels of cytosolic calcium is unlikely to be involved in cardioprotection in immature heart. This is because we found higher concentrations of propofol which depress function (presumably by inhibiting calcium channels) are not protective. On the contrary, 10 *μ*g/mL propofol seemed to worsen structural damage (Figures 3 and 4) as shown by the number of dense material deposits in mitochondria and the degree of mitochondrial swelling [38]. The degree of clumping of nuclear chromatin, the degree of intracellular edema, and the degree of appearance of I-band in myocyte present a degree of cardiomyocyte damage (Table 2 and Figures 3 and 4).

Earlier work has reported that the cardioprotective effect in the adult heart is due to inhibition of MPTP [11]. This is consistent with our finding that inhibition of MPTP using CsA also protected the immature heart and this protection was similar to protection by propofol when used at 2 *μ*g/mL. We previously reported that hypothermia and hyperkalemia protect the heart from I/R injury in mature guinea pig hearts, in which we observed that both hypothermia and hyperkalemia dramatically reduced lactate release as from immediately starting reperfusion [39]. In the present study, 2 *μ*g/mL of propofol and CsA showed similar changes in lactate release without significant differences to control during the first 3 minutes and with significant decreases at 5 minutes after reperfusion. This finding may indicate a similar mechanism of protection between propofol and CsA and probably means a different mechanism compared to hypothermia and hyperkalemia. The observation of LVEDP at the end of ischemia without significant differences among control and 2 *μ*g/mL of propofol and CsA may support this speculation because it was significantly reduced by hypothermia and hyperkalemia in our previous work. Since CsA and propofol are established as MPTP inhibitor by numerous previous works, we believe that the demonstrated myocardial protection by propofol in this study was at least partly due to inhibition of MPTP opening.

It is known that swelling or edema of mitochondria is an important finding of I/R injury in studies of myocardial protection. Previous studies determined the degree of swelling by evaluating the matrix clarity and separation of cristae in mitochondria, and we followed their way of assessment in this study. Although our protocol did not bring severe edema to mitochondria in control group, mitochondria with 2 *μ*g/mL of propofol showed even less edema than control group at the end of the reperfusion period. Increase of dense material deposits (size and numbers), which is caused by calcium overloading to the mitochondria, is also an important marker of mitochondrial damage during I/R [38, 40]. The structure of mitochondria, including these deposits and cristae, was well preserved with 2 *μ*g/mL of propofol at the end of protocol. The I-band is another finding of I/R injury, which increases in cardiomyocytes with moderate to severe damage by I/R [27]. In the present study, 2 *μ*g/mL of propofol showed mild changes to the cardiomyocytes which are potentially reversible. Although cardiomyocytes in the other 2 groups were identified as moderately (not severely) damaged in histopathological examination, they were judged by an expert pathologist as irreversible changes.

Previous studies demonstrated negative inotropic effect of propofol with decreased intracellular calcium in the myocardium. The effect is known to depend on its concentration [41]. Our study showed that propofol at 10 *μ*g/mL showed significant negative inotropic changes before ischemia and more importantly, it did not have protective effect against I/R injury, which was confirmed not only by functional recovery but also in histopathological examination. Our results indicate potentially harmful effects of high concentration of propofol for immature heart based on histopathological investigation. High concentrations of propofol can cause decreased production of ATP and inhibition of cardiac L-type calcium current (which causes low level of intracellular calcium) before, during, and immediately after ischemia [33, 42, 43] and may introduce an exaggerated calcium influx during the following period (same mechanism as calcium paradox). Excessively increased dense material deposit, which consists of calcium in mitochondria [44], seen in the heart treated with 10 *μ*g/mL of propofol might reflect this mechanism. Thus, the potential mechanism of harmful effects of high concentration of propofol is speculated. MPTP inhibition is difficult under conditions of low adenine nucleotides and excessive calcium. Further investigations are necessary to elucidate the details.

## 5. Conclusion

This study shows that propofol at clinically relevant concentrations can protect immature hearts from I/R injury and that this effect is dose dependent where optimal protection is similar to what is seen when inhibiting the MPTP. In contrast to the beneficial effect of clinically relevant concentration of propofol, high dose of propofol can be harmful to the immature heart.

## Figures and Tables

**Table:** (a) Changes in developed and end diastolic pressures

	Developed pressure (mmHg)		End diastolic pressure (mmHg)		
	Equilibrium	End of protocol	Equilibrium	End of ischemia	End of protocol
Control	88.4 ± 10.7	44.2 ± 6.3	4.3 ± 0.8	30.6 ± 3.2	16.3 ± 2.4
Propofol (*μ*g/mL)					
1	83.6 ± 5.2	60.8 ± 6.8^∗1^	5.2 ± 0.7	28.9 ± 2.6	6.6 ± 1.6
2	72.2 ± 5.8	56.6 ± 6.0^∗2^	4.3 ± 1.2	38.7 ± 3.0	6.7 ± 4.3
4	75.6 ± 4.0	47.8 ± 3.9	5.0 ± 0.5	28.5 ± 3.3	7.4 ± 3.0
10	72.2 ± 4.7	36.5 ± 4.4	4.2 ± 1.0	31.3 ± 4.8	12.0 ± 3.6
Cyclosporine A	76.7 ± 6.7	52.9 ± 4.0^∗2^	4.0 ± 0.7	36.2 ± 3.8	9.1 ± 3.0

Data are presented as mean ± SE. ^∗1^ *P* < 0.05 versus control and 10 *μ*g/mL of propofol; ^∗2^ *P* < 0.05 versus 10 *μ*g/mL of propofol.

**Table:** (b) Changes in coronary flow following ischemia and reperfusion

	Coronary flow (mL/min)		Recovery (%)
	Equilibrium	End of protocol	
Control	11.0 ± 0.8	5.7 ± 0.5	52.5 ± 2.9
Propofol (*μ*g/mL)			
1	11.4 ± 0.9	7.8 ± 0.5	69.8 ± 4.1*
2	10.2 ± 0.7	7.0 ± 0.4	69.1 ± 1.8*
4	11.9 ± 0.5	6.9 ± 0.7	57.5 ± 5.5
10	10.3 ± 0.4	5.8 ± 0.5	57.0 ± 4.1
Cyclosporine A	11.3 ± 0.8	7.1 ± 0.5	61.6 ± 2.1^a^

Data are presented as mean ± SE. *P < 0.05 versus control; ^a^ *P* = 0.08 versus control.

**Table 2:** Histological changes following ischemia and reperfusion.

	Light microscopy			Electron microscopy			
	Clumping of nuclear chromatin	Intracellular edema	Interstitial edema	I-band in myocyte	(Mitochondrial changes)		
					Dense material deposit	Clarity of matrix	Deformation of cristae
Control	2	2	3	1	2	2	1
2 *μ*g/mL Propofol	1	1	1	0	1	1	1
10 *μ*g/mL Propofol	3	2	2	1	3	3	2

The scale of 0 to 4 represents the degrees of each morphological change: 0, normal; 1, trivial; 2, mild; 3, moderate; and 4, severe.
